# Nitric Oxide and Abscisic Acid Mediate Heat Stress Tolerance through Regulation of Osmolytes and Antioxidants to Protect Photosynthesis and Growth in Wheat Plants

**DOI:** 10.3390/antiox11020372

**Published:** 2022-02-12

**Authors:** Noushina Iqbal, Zebus Sehar, Mehar Fatma, Shahid Umar, Adriano Sofo, Nafees A. Khan

**Affiliations:** 1 Department of Botany, Jamia Hamdard, New Delhi 110062, India; naushina.iqbal@gmail.com; 2 Plant Physiology and Biochemistry Laboratory, Department of Botany, Aligarh Muslim University, Aligarh 202002, India; seharzebus5779@gmail.com (Z.S.); meharfatma30@gmail.com (M.F.); 3 Department of European and Mediterranean Cultures: Architecture, Environment, Cultural Heritage (DiCEM), University of Basilicata, Via Lanera, 75100 Matera, Italy

**Keywords:** abscisic acid, glycine-betaine, nitric oxide, proline, total soluble sugar, trehalose

## Abstract

Nitric oxide (NO) and abscisic acid (ABA) play a significant role to combat abiotic stress. Application of 100 µM sodium nitroprusside (SNP, NO donor) or ABA alleviated heat stress effects on photosynthesis and growth of wheat (*Triticum aestivum* L.) plants exposed to 40 °C for 6 h every day for 15 days. We have shown that ABA and NO synergistically interact to reduce the heat stress effects on photosynthesis and growth via reducing the content of H_2_O_2_ and thiobarbituric acid reactive substances (TBARS), as well as maximizing osmolytes production and the activity and expression of antioxidant enzymes. The inhibition of NO and ABA using c-PTIO (2-4 carboxyphenyl-4,4,5,5-tetramethylimidazoline-1-oxyl-3-oxide) and fluridone (Flu), respectively, reduced the osmolyte and antioxidant metabolism and heat stress tolerance. The inhibition of NO significantly reduced the ABA-induced osmolytes and antioxidant metabolism, exhibiting that the function of ABA in the alleviation of heat stress was NO dependent and can be enhanced with NO supplementation.Thus, regulating the activity and expression of antioxidant enzymes together with osmolytes production could act as a possible strategy for heat tolerance.

## 1. Introduction

The rise in temperature beyond the plants’ optimal requirement for growth is one of the most serious abiotic stressors causing irreparable harm to the development of plants [1,2]. Climate change is expected to significantly affect agriculture and food security [3]. It has been estimated that a gradual expected rise in the global temperature may influence plant productivity prominently, particularly in the next decades [4]. The average world temperature over the next 20 years may reach or exceed 1.5 °C of warming. At 2 °C of global warming, heat extremes would more often reach critical tolerance thresholds for agriculture and health according to the Intergovernmental Panel on Climate Change [5]. Among the alterations, excessive temperature stress during the crop reproductive phase has a likely detrimental influence on agricultural productivity [6,7]. It has been shown that high temperatures adversely affected plant molecular, biochemical, and physiological characteristics [8,9,10,11,12,13]. Heat stress causes an oxidative burst, membrane lipid peroxidation, pigment bleaching, protein degradation, enzyme inactivation, and macromolecule damage in plants [2,14,15,16]. It influences cell differentiation and elongation, cytoskeleton degradation [2,17], and the activity of chloroplasts [18].

The plant’s reaction to heat stress depends on its developmental stage and the severity of the stress [19,20]. Devasirvatham et al. [21] showed that higher temperaturestress during anthesis resulted in more aborted and deformed buds, while heat stress during the vegetative stage negatively influenced plant’s growth, morphology, and nutrient absorption. However, the optimal temperature varies across compartments within the cell and between species within the genus [22]. A recent research on 12 cultivars of *Oryza sativa* has shown a negative and differential impact of high night temperature on plant metabolism [23]). As a result, plant scientists are focused on discovering signaling molecules that have the potential to shield plants against the negative impacts of climate change [24,25,26]. Exogenous application of osmoprotectants, phytohormones, signaling molecules, and trace elements has demonstrated to benefit plants in high temperature environments, owing to their growth-promoting and antioxidant properties [27,28,29,30].

Nitric oxide (NO) is gaining growing interest from the plant science community due to its participation in the resistance to diverse plant stress situations; however, its effects on heat stress tolerance are still debatable. It is a free radical gaseous molecule that has been shown to be involved in diverse biological functions in plants [31]. Nitric oxide is an excellent diffusible chemical messenger in plant signaling due to the fact that it is a small diatomic molecule with a short half-life and lacks charge [32]. Over time, evidence developed that NO plays a vital function in a variety of plant physiological processes, including seed germination [33,34], senescence and maturation [35], and multiple abiotic stress responses [36,37]. Several researchers have focused on explaining the critical function of NO in modulating different plant hormone-mediated growth and stress responses [12,38,39,40,41]. NO might act as a secondary messenger for the other protective compound. Diao et al. [42] found that NO was induced by spermidine in tomato under chilling stress and was responsible for chilling tolerance because its inhibition inhibited tolerance. NO could be a common signaling component for various elicitors and phytohormones, including ABA [43]; therefore, its crosstalk is important. Apart from NO, abscisic acid (ABA) also plays a pivotal role in the plant’s response to various abiotic stresses, such as heat, water deficit, and salinity, and regulates stomatal closure and production of several acclimation proteins [44,45]. Teplova et al. [46] reported that ABA content was increased in response to heat exposure in tobacco plant. Gong et al. [47] have reported that thermotolerance induced by ABA supplementation in maize was mediated by Ca^2+^ and associated with antioxidant systems. ABA-responsive marker genes were implicated in heat tolerance which include *AREB1* (ABA response element-binding protein 1), *LTP1* (lipid transfer protein 1), and *DREB3* (dehydration-responsive element-binding protein 3), and were also increased in *NtDOG1L-T* transgenic lines in tobacco under heat stress to induce heat tolerance [48].

The interaction between NO and ABA under heat stress has been reviewed [49]. Nitric oxide triggers antioxidant gene expression or activates antioxidant enzymes through post-translational modifications for stress tolerance [50]. In wheat, Alnusairi et al. [51] reported that treatment with NO increased the accumulation of proline and soluble sugars and also upregulated the antioxidant system in order to reduce salt-induced oxidative damage on membranes. Nitic oxide was found to decrease the superoxide dismutase/ascorbate peroxidase (SOD/APX) ratio to eliminate Al-induced excess hydrogen peroxide (H_2_O_2_) and malondialdehyde (MDA) to prevent programmed cell death in peanut root tips [52]. Ahmad et al. [53] reported the role of NO in salt tolerance via enhancing the expression of *SOD*, *APX*, and glutathine reductase (*GR*), together with increased accumulation of osmolytes. 

Similarly, Guan et al. [54] found ABA role in regulating the expression of the *Cat1* gene during late maize embryogenesis under osmotic stress in maize. Zhang et al. [55] found a C_2_H_2_-type zinc finger protein, *ZFP36*, for ABA-induced antioxidant defense, which resulted in rice tolerance to water stress and oxidative stress. ABA-induced antioxidant defense in maize was responsible for drought and heat tolerance via HSP70 [56]. ABA was required for expression of the *P5CS* gene during salt stress in *Arabidopsis* [57]. Studies have reported the role of proline, glycine betaine (GB), and calcium in thermotolerance and their induction by ABA [47,58,59] and NO [60]. Nitric oxide was found to increase the synthesis of ABA in wheat seedlings exposed to salt stress and acted downstream of ABA in ABA-induced proline accumulation [61]. Nitric oxide is also reported to enhance proline accumulation under salt stress [62] and osmotic stress [63]. Reports suggest that NO was required for the ABA-dependent proline formation under salt stress [61]. Li et al. [64] found that H_2_S increases trehalose accumulation under heat stress, which helps heat tolerance. 

Wheat is an economically important crop that provides nutrition to the population. It is an important cereal crop and rich source of proteins, vitamins, and dietary fibers, as well as a source of food for many developing countries worldwide [65]. It is the largest food crop, producing 776.1 million tons of grains to add to the world crop production [66]. The demand for wheat is increasing and a 2.0% annual increase is predicted [67]; however, is threatened by heat and water stress [68]. Heat stress reduces its photosynthetic potential and growth [69,70] and affects wheat’s physiological, biological, and biochemical processes [71]. The present study explored the interaction between NO and ABA in mitigating heat stress in the wheat crop through their impact on osmolytes accumulation and the activity and expression of antioxidant enzymes. 

In this research, we investigated their individual and interactive effect under heat stress in wheat through regulation of osmolytes and antioxidants. As the hormonal action is interrelated through a cascade of signaling molecules, we have shown the coordination of NO and ABA in heat stress tolerance, with major emphasis on osmolytes and antioxidants. Such a study, where the importance of antioxidants and osmolytes is shown in photosynthetic protection through ABA and NO involvement, has not been done.

## 2. Materials and Methods

### 2.1. Plant Material and Growth Conditions

Seeds of wheat (*Triticum aestivum* L.) cultivar WH542 obtained from the National Seeds Corporation, New Delhi, India, were surface sterilized with 0.01% HgCl_2_ followed by repeated washing with double-distilled water. The seeds were then sown in acid-washed purified sand in pots of 23-cm in diameter. The pots were placed in an environmental growth chamber (Khera-Instruments, New Delhi, India) with day/night temperatures at 25/18 °C, 12 h photoperiod (PAR 300 µmol m^−2^ s^−1^), and relative humidity of 65 ± 5%. Two plants per pot were maintained and 150 mL of full-strength Hoagland’s nutrition solution was applied alternate days. After seedling emergence (10 days after sowing, DAS), the plants were subjected to temperature stress treatment by keeping them at 40 °C for 6 h every day for 15 days with all other growth conditions remaining the same, after which they were allowed to recover at the optimal temperature (25 °C) and grown for the experimental period. The control plants were maintained at 25 °C throughout the experimental growth period (30 days). To investigate the role of NO and ABA in mitigating the adverse effects of high temperature stress, sodium nitroprusside (SNP, NO donor) and ABA, each at 100 µM concentration, were applied on the foliage of plants either alone or in combination, with a hand sprayer at 10 DAS; i.e., before heat stress. A 0.5% teepol as surfactant was added with the treatments spray including control which received spray of double distilled water. To understand the contribution of the effect of NO and ABA, their inhibitors cPTIO (2-4 carboxyphenyl-4,4,5,5-tetramethylimidazoline-1-oxyl-3-oxide, NO scavenger) at 100 µM and fluridone (Flu, ABA biosynthesis inhibitor) at 80 µM, respectively, were applied at 10 DAS with NO/ABA treatments. The concentrations of SNP and ABA were determined based on the study of Iqbal et al. [70] and Wu et al. [72], respectively. The spray volume of the chemicals was approximately 30 mL. The treatments were arranged in a randomly blocked design with four replicates (*n* = 4) for each treatment. At 30 DAS, plants were sampled for different measurements.

### 2.2. Determination of Growth Characteristics 

Plants were uprooted and washed gently under running tap water to remove the adhering sand. Plants were then blotted with soft paper towel to remove any free moisture. Plant length was measured on a meter scale. Fresh weight of plants was determined using an electronic balance, while dry weight of the plants was recorded after drying the sample in a hot air oven at 80 °C for 72 h until reaching a constant weight. Leaf area was measured by using leaf area meter (LA211, Systronic, New Delhi, India).

### 2.3. Photosynthetic Characteristics Measurements

The fully expanded third leaf of each treatment was taken for gas exchange measurements. An infrared gas analyzer (CID-340, Photosynthesis System, Bio-science, Washington, DC, USA) was used for measurements of net photosynthesis (P_N_), stomatal conductance (gs), and intercellular CO_2_ concentration (Ci). The details of the method are given in Supplementary File S1. Chlorophyll content was measured with a SPAD chlorophyll meter (SPAD 502 DL PLUS, Konica Minolta, Japan). 

The maximal efficiency of photosystem II (PSII), as given by Fv/Fm, was determined with a chlorophyll fluorometer (Junior-PAM, Heinz Walz, GmbH, Effeltrich, Germany). The process is detailed in Supplementary File S1.

Ribulose 1,5-bisphosphate carboxylase/oxygenase (Rubisco, EC 4.1.1.39) activity was spectrophotometrically determined by adopting the method of Usuda [73]. The details are given in Supplementary File S1.

### 2.4. Determination of H_2_O_2_ and Thiobarbituric Acid Reactive Substance (TBARS) Content

Content of H_2_O_2_ was determined by adopting the method of Okuda et al. [74]. Lipid peroxidation was determined by measuring the content of thiobarbituric acid reactive substance (TBARS), as described by Dhindsa et al. [75]. The details of the procedures are given in Supplementary File S1.

### 2.5. Abscisic Acid Content

The ABA content was determined by adopting the method of Hung and Kao [76], with slight modifications. The details of the procedure are mentioned in Fatma et al. [77]. ABA was determined spectrophotometrically at 405 nm with an ABA immunoassay detection kit (model PGR-1; Sigma-Aldrich, St. Louis, MO, USA).

### 2.6. Determination of NO Generation

Nitric oxide generation was confirmed by estimating the nitrite content by adopting the method of Zhou et al. [78], with slight modifications. The details of the method are presented in Gautam et al. [12]. The absorbance of the reaction mixture was read at 540 nm and NO content was estimated from a calibration curve plotted using sodium nitrite as standard.

### 2.7. Assay of Activity of Antioxidant Enzymes

The top-most leaves were used to get 200 mg of fresh leaf tissue and it was rapidly grounded in ice-cold extraction buffer and centrifuged at 15,000× *g* for 20 min at 4 °C. The supernatant was used for the assay of enzymes. Protein was estimated following the Bradford [79] method utilizing bovine serum albumin as a protein standard. Complete detail of the extraction buffer and method is given in Supplementary File S1.

The Beyer and Fridovich [80] and Giannopolitis and Ries [81] methods were used for determining superoxide dismutase (SOD) activity in the protein extracts based on inhibition of the photochemical reduction of nitro blue tetrazolium (NBT). The Aebi [82] method with slight modification was adopted for observing the disappearance of H_2_O_2_ at 240 nm for determination of the catalase (CAT) activity. For the assay of ascorbate peroxidase (APX), the Nakano and Asada [83] method was adopted, and the glutathione reductase (GR) activity was estimated following the method of Foyer and Halliwell [84]. The oxidation of nicotinamide adenine dinucleotide phosphate (NADPH) at 340 nm was monitored in the presence of glutathione (GSH). The details of method adopted for measuring the activity of antioxidant enzymes are given in Supplementary File S1.

### 2.8. Determination of Proline Content

Proline content was estimated in the leaves using the method of Bates et al. [85]. Fresh leaf tissue (300 mg) was homogenized in 3.0 mL of 3% sulphosalicylic acid, and the homogenate was centrifuged at 11,500× *g* for 12 min. The supernatant filtrate was added to a test tube with 2.0 mL acid ninhydrin and 2.0 mL glacial acetic acid and incubated in water bath at 100 °C for 1 h. Later, 4.0 mL toluene was added to the reaction mixture and mixed vigorously with stirrer for 20–30 s, and then left to stand for 5–10 min. The absorbance of the reddish pink upper phase was measured on a spectrophotometer at 520 nm using L-proline as a standard.

### 2.9. Determination of Glycine Betaine Content

The estimation of glycine betaine (GB) in leaves was based on the formation of the betaine–periodite complex by adopting the method of Grieve and Grattan [86]. The details of the procedure are given in Syeed et al. [87].

### 2.10. Estimation of Total Soluble Sugar and Trehalose Content

The method of Xu et al. [88] and Gautam et al. [13] were adopted for estimation of total soluble sugar content. Trehalose content in leaves was determined following the method described by Li et al. [64], with some modifications. The details of the procedure done are given in Supplementary File S1.

### 2.11. Quantitative RT-PCR Analysis 

Total RNA extraction for gene expression was done in fresh leaves (100 mg) of four biological samples of plants subjected to different treatments using Trizol reagent according to the instructions given in the kit (Ambion, Life Technologies, Austin, TX, USA). The specific primers were designed to assess gene expression. All reactions were performed as three biological replicates (with four technical replicates of each), using gene-specific primers and actin primers as an internal control. The results were presented as the expression of the gene of interest in relation to the internal control in the treated sample compared with corresponding values in the untreated control.The primer pairs used for quantitative RT-PCR and the details of the procedure are provided in Supplementary File S1.

### 2.12. Statistical Analysis

The data were statistically analyzed using Analysis of variance (ANOVA) with the help of software SPSS 17.0 for Windows (SPSS Inc., Chicago, IL, USA). Data were presented as the mean ± SE (*n* = 4) and the significant differences were identified on the basis of the Tukey test at *p* < 0.05. 

## 3. Results

We tested the individual and combined effect of NO and ABA in heat stress alleviation. The combined NO and ABA treatment under heat stress was also subjected to 100 µM cPTIO or 80 µM fluridone at 30 DAS.

### 3.1. Effect of NO and ABA on Growth Parameters under Heat Stress 

**Figure 1:**
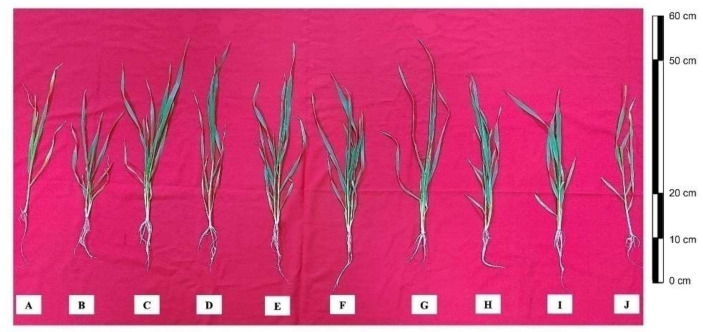
Leaves of wheat (*Triticum aestivum* L. var. WH 542) plants were treated with 100 µM SNP and/or ABA in the presence (40 °C) or absence (25 °C) of heat stress at 30 days after sowing (DAS). The combined NO and ABA treatment under heat stress was also subjected to 100 µM cPTIO and/or 80 µM fluridone (Flu) at 30 DAS. (A) control, (B) heat stress, (C) SNP, (D) ABA, (E) SNP + ABA, (F) HT + SNP, (G) HT + ABA, (H) HT + SNP + ABA, (I) HT + SNP + ABA + Flu, and (J) HT + SNP + ABA + cPTIO. ABA, abscisic acid; cPTIO, 2-4-carboxyphenyl-4,4,5,5-tetramethylimidazoline-1-oxyl-3-oxide; Flu, fluridone; HT, heat stress; SNP, sodium nitroprusside.

The indices of growth—plant length, leaf area, and plant fresh and dry weights—were analyzed under heat stress. The relative data are shown in Table 1. Heat stress decreased plant length by 21.3%, leaf area by 32.7%, plant fresh weight by 25.0%, and plant dry weight by 51.6% in comparison to the control plants. Individual application of NO and ABA increased the indices of growth. However combined application of NO and ABA maximally increased these parameters by 17.1%, 42.7%, 28.6%, and 58.3%, respectively, in comparison to the control plants. Supplementation of NO/ABA exhibited an increase in growth indices of plant by decreasing the negative effects caused by heat stress. Moreover, the combined application of NO and ABA significantly alleviated the effect of heat stress and increased plant length by 37.8%, leaf area by 76%, plant fresh weight by 38%, and plant dry weight by 177% in comparison to the heat-stressed plants. Both cPTIO and Flu inhibited the increase observed in growth parameters with NO and ABA under heat stress. Supplementation of Flu to the plants receiving NO and ABA under heat stress alleviated the effect of heat stress and increased these parameters by 27%, 49.1%, 28.6%, and 134%, respectively, in comparison to the heat-stressed plants. Moreover, cPTIO treatment to plants receiving NO and ABA under heat stress showed less alleviation of heat stress and increased these parameters by 8.1%, 7.1%, 4.8%, and 20.5%, respectively, compared to the plants exposed to heat stress. The response of plants to inhibitors exhibited that the action of ABA in heat tolerance was through NO action (Table 1; Figure 1).

### 3.2. Impact of Heat Stress on Photosynthetic Characteristics and Involvement of NO and ABA in Inhibiting Heat-Induced Photosynthetic Reduction

Table 2 shows that heat stress reduced P_N_ by 44.6%, gs by 28.4%, Ci by 43.6%, Chl by 37.0%, Fv/Fm by 16.7%, and Rubisco by 35.2% compared to plants grown under control conditions. Individual application of NO and ABA under non-stress conditions increased the studied photosynthetic parameters but maximal increase occurred with their combined application. In the same line, NO and ABA effectively reduced the heat stress effects on photosynthetic parameters. The increase in photosynthetic parameters due to NO and ABA under normal conditions and alleviation under heat stress were greater with NO than ABA. Application of NO reduced the inhibitory effect of heat stress on photosynthetic parameters to the level of control. 

The interaction of NO and ABA in the enhancement in photosynthesis parameters was further investigated using their inhibitors. The combined NO and ABA treatment under heat stress together with Flu (ABA inhibition) resulted in bringing the photosynthetic parameters significantly equal to heat stress plus NO treatment, suggesting that inhibition of ABA did not affect NO-mediated tolerance, an indication of downstream NO signaling. On the other hand, cPTIO treatment to the plants receiving NO and ABA under heat stress decreased the photosynthetic parameters compared to the control (Table 2).

The photosynthetic parameters noted in NO plus heat stress treatment were significantly equal to heat stress plus NO plus ABA plus Flu treatment. Instead, when NO was inhibited by cPTIO, we observed a much more significant decrease in photosynthetic characteristics, suggesting the action of ABA being dependent on NO or ABA functions upward of NO in heat stress signaling (Table 2).

### 3.3. ABA Requires NO Action for Reducing Oxidative Stress in Wheat Plants Exposed to Heat Stress

**Figure 2:**
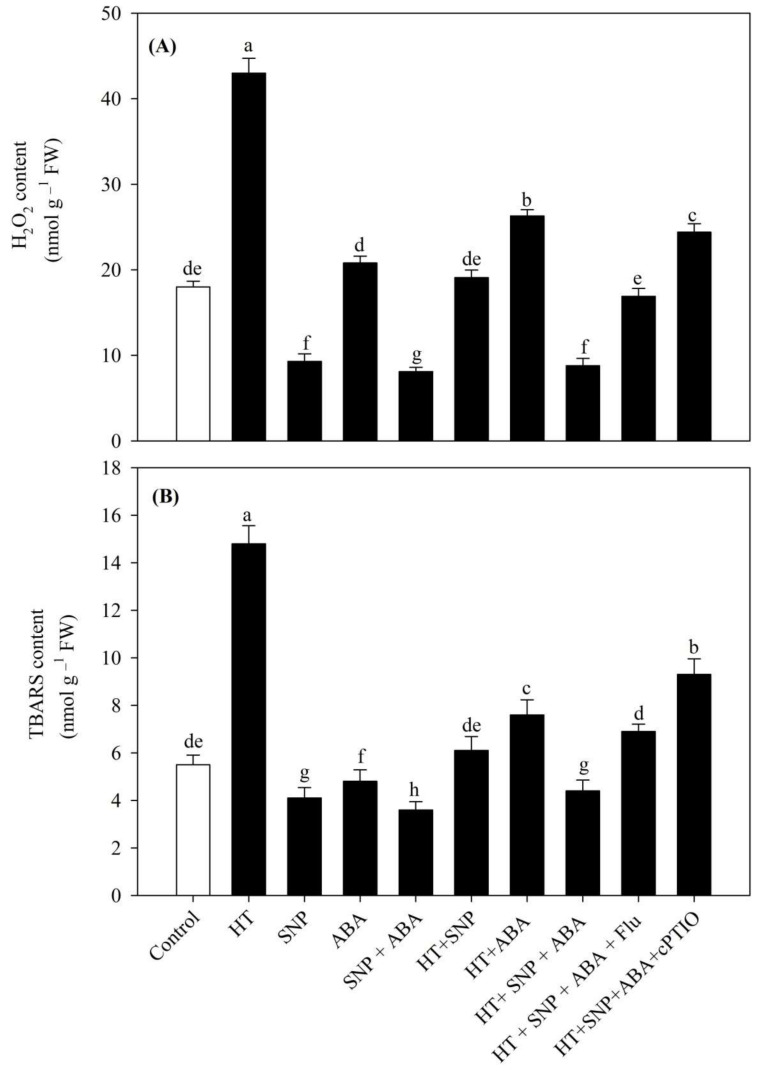
(**A**) H_2_O_2_ and (**B**) thiobarbituric acid reactive substances (TBARS) content of wheat (*Triticum aestivum* L. var. WH 542) leaves treated with 100 µM SNP and/or ABA in the presence (40 °C) or absence (25 °C) of heat stress at 30 days after sowing (DAS). The combined NO and ABA treatment under heat stress was also subjected to 100 µM cPTIO and/or 80 µM fluridone at 30 DAS. Data are presented as the treatment mean ± SE (*n* = 4). Data followed by the same letter are not significantly different by Tukey test at *p* < 0.05. ABA, abscisic acid; cPTIO, 2-4-carboxyphenyl-4,4,5,5-tetramethylimidazoline-1-oxyl-3-oxide; Flu, fluridone; HT, heat stress; SNP, sodium nitroprusside.

Figure 2 shows that heat stress significantly induced high oxidative stress, which was related to the high H_2_O_2_ (+138.9%) and TBARS (+169.1%) content compared to the control.

NO treatment to the plants reduced oxidative stress in terms of the H_2_O_2_ and TBARS content by 48.3% and 25.4%, whereas ABA increased the H_2_O_2_ content by 15.5% and decreased the TBARS content by 12.7%, in comparison to control under no stress conditions. However, plants supplemented with NO and ABA showed the maximum reduction in H_2_O_2_ and TBARS content by 55.0% and 34.5%, compared to the control. Under heat stress, treatment of NO alongwith ABA decreased the heat-induced H_2_O_2_ and TBARS content by 79.5% and 72.3%, respectively, in comparison to the plants grown under heat stress. Application of Flu to plants receiving both ABA and NO under heat stress showed a significant decrease in oxidative stress markers, which was significantly equal to the control and also to the heat stress plus SNP treatment, suggesting that ABA inhibition has not affected a NO-mediated reduction in H_2_O_2_ and TBARS content. Contrarily, cPTIO alongwith combined treatment of NO and ABA plus heat showed an increase in the biomarkers of oxidative stress compared to the control. Thus, when the interaction effect was studied, it was found that the presence of NO has a major role in reducing oxidative stress compared to ABA and thus is responsible for the maintenance of photosynthesis under heat stress. 

### 3.4. Impact of Heat Stress on NO and ABA Content

**Figure 3:**
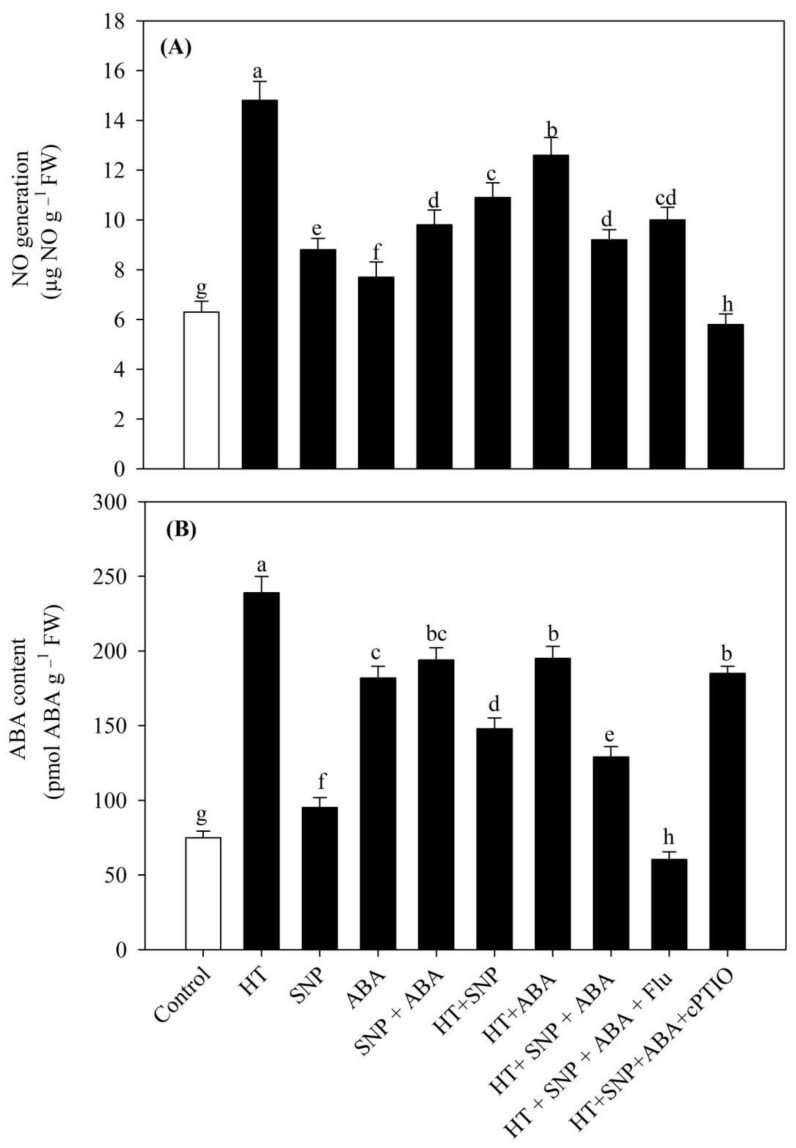
(**A**) Nitric oxide generation and (**B**) ABA content of wheat (*Triticum aestivum* L. var. WH 542) leaves treated with 100 µM SNP and/or ABA in the presence (40 °C) or absence (25 °C) of heat stress at 30 days after sowing (DAS). The combined NO and ABA treatment under heat stress was also subjected to 100 µM cPTIO and/or 80 µM fluridone at 30 DAS. Data are presented as the mean ± SE (*n* = 4). Data followed by the same letter are not significantly different by Tukey test at *p* < 0.05. ABA, abscisic acid; cPTIO, 2-4-carboxyphenyl-4,4,5,5-tetramethylimidazoline-1-oxyl-3-oxide; Flu, fluridone; HT, heat stress; SNP, sodium nitroprusside.

Figure 3 shows the content of NO and ABA in leaves of wheat plants exposed to heat stress. Heat stress increased the NO generation and ABA content by 135.1% and 218.6%, in comparison to control plants. Under no stress conditions, individual or combined treatment of NO and ABA increased both the NO generation and ABA content and maximally when in combination compared to the control. Contrarily, under heat stress, plants treated with both NO and ABA showed a reduction in ABA and NO content by 37.8% and 46.1% compared to the heat stressed plants. Inhibition of ABA and/or NO by their biosynthesis inhibitor, such as Flu and/or cPTIO, reduced the content of NO generation by 32.4% and 60.8% and ABA content by 74.7% and 22.6%, respectively, compared to the heat-stressed plants.

### 3.5. The Combined Application of NO and ABA Stimulated Antioxidant Enzyme Activity under Heat Stress

Table 3 shows that heat stress increased the activity of antioxidant enzymes such as SOD, CAT, APX, and GR by 54.7%, 19.6%, 68.2%, and 62.4%, respectively, in comparison to control plants. Individual application of NO and ABA also increased the antioxidant enzyme activity, which was maximally increased when both ABA and NO were applied together under no stress.

Under heat stress, treatment of NO alongwith ABA stimulated the increase in activity of SOD by 74%, CAT by 42%, APX by 121.6%, and GR by 72%, relative to the high-temperature-treated plants. However, upon application of Flu to NO plus ABA under heat stress, the activity of these antioxidant enzymes was reduced compared to NO plus ABA treatment under heat stress. A further reduction in the activity of the antioxidant enzymes occurred when cPTIO was applied to a combined NO and ABA treatment under heat stress and was significantly equal to the heat stress treatment.

### 3.6. Osmolytes Accumulation Increased under Heat Stress Maximally with Combined NO and ABA Supplementation

**Figure 4:**
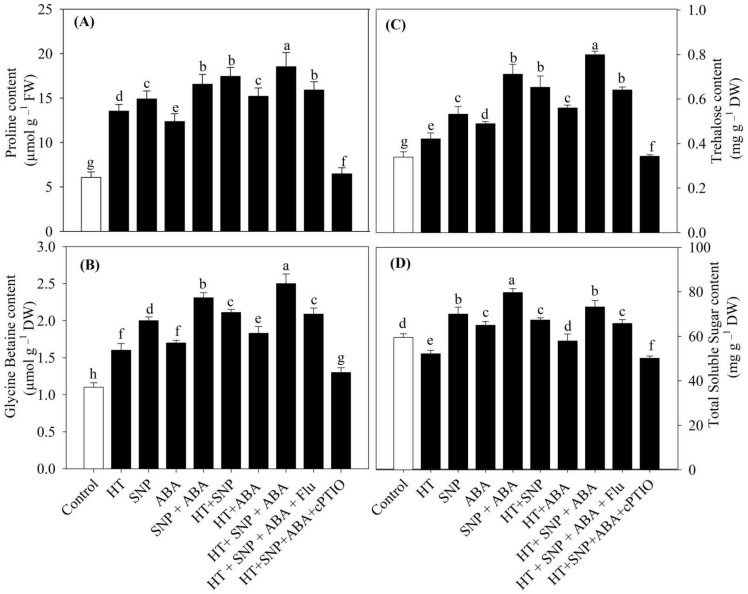
(**A**) Proline, (**B**) glycine betaine, (**C**) trehalose, and (**D**) total soluble sugar of wheat (*Triticum aestivum* L. var. WH 542) leaves treated with 100 µM SNP and/or ABA in the presence (40 °C) or absence (25 °C) of heat stress at 30 days after sowing (DAS). The combined NO and ABA treatment under heat stress was also subjected to 100 µM cPTIO and/or 80 µM fluridone at 30 DAS. Data are presented as the treatment mean ± SE (*n* = 4). Data followed by the same letter are not significantly different by Tukey test at *p* < 0.05. ABA, abscisic acid; cPTIO, 2-4-carboxyphenyl-4,4,5,5-tetramethylimidazoline-1-oxyl-3-oxide; Flu, fluridone; HT, heat stress; SNP, sodium nitroprusside.

Osmolytes accumulation increased under heat stress due to the activation of stress signaling pathways and minimized oxidative damage and growth restriction with the increase in photosynthesis efficiency. We studied proline, GB, trehalose, and total soluble sugar content for their role in defense against heat stress (Figure 4). Heat stress triggered proline accumulation and increased it by 21%, GB by 45.5%, and trehalose by 24%, but decreased the total soluble sugar content by 12.3%, relative to the control. Treatment with individual NO and ABA led to even more substantial increase in osmolyte content and maximally when plants were treated with both NO and ABA together. Under heat stress, application of NO and ABA resulted in a significant increase in osmolyte accumulation, such as the proline content by 113.3%, GB content by 56.2%, trehalose content by 90%, and total soluble sugar content by 40.2%, relative to the heat-stressed plants. Moreover, treatment of Flu to the plants receiving NO and ABA under heat stress further increased the osmolyte content, while addition of cPTIO to the plants supplemented with NO and ABA under heat stress showed a reduction in the respective parameters in comparison to the high-temperature-treated plants. The reduction in the content of osmolytes occurred with both NO and ABA inhibition when compared to their combined application under heat stress; however, Flu caused a lesser reduction compared to cPTIO, indicative of ABA requiring NO for its action.

### 3.7. Application of NO and ABA on Antioxidant Enzyme Gene Expression under Heat Stress

**Figure 5:**
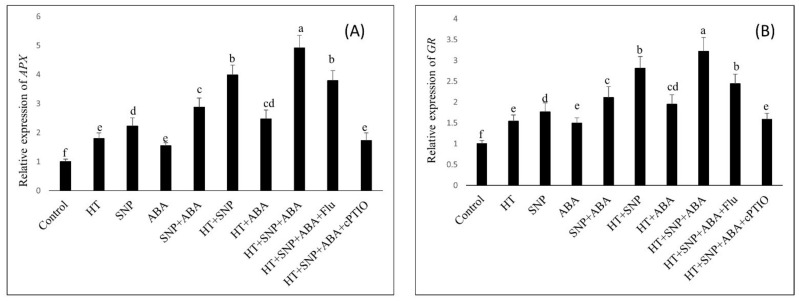
Relative expression of the genes (**A**) *APX* and (**B**) *GR* of wheat (*Triticum aestivum* L. var. WH 542) leaves treated with 100 µM SNP and/or ABA in the presence (40 °C) or absence (25 °C) of heat stress at 30 days after sowing (DAS). The combined NO and ABA treatment under heat stress was also subjected to 100 µM cPTIO and/or 80 µM fluridone (Flu) at 30 DAS. Results are represented relative to the total of the respective control (control, 1). Data are presented as the mean ± SE (*n* = 4). Data followed by the same letter are not significantly different by Tukey test at *p* < 0.05.ABA, abscisic acid; cPTIO, 2-4-carboxyphenyl-4,4,5,5- tetramethylimidazoline-1-oxyl-3-oxide; Flu, fluridone; HT, heat stress; SNP, sodium nitroprusside.

Exogenous application of NO with ABA increased the activity of antioxidant enzymes under heat stress, so we tested the changes in the expression level of *APX* and *GR* genes by the exogenous application of NO with ABA under heat stress, and also with their inhibitors as 100 µM cPTIO and/or 80 µM fluridone. The expression of *APX* and *GR* was upregulated with the combined treatments of NO and ABA under heat stress compared to the unstressed wheat plants. However, compared to Flu, cPTIO application to combined NO plus ABA under heat stress caused higher downregulation of antioxidant enzyme gene expression, suggesting that the effect of ABA was mediated via NO (Figure 5).

## 4. Discussion

Increased temperatures, above what is required for plant growth, significantly reduces photosynthesis and impedes growth and yield [1,89,90]. According to the IPCC [5], global warming of 1.5 °C and 2 °C will be exceeded during the 21st century unless emission of greenhouse gases is controlled. Plants exposed to heat stress exhibit overproduction of ROS that cause lipid per-oxidation, DNA damage, protein oxidation, and cell apoptosis [91,92,93,94,95]. However, these ROS function as a signaling molecule that confers plants to acclimate and adapt to abiotic stresses [96]. One such mechanism of adaptation is the activation of antioxidative enzymes in the plants exposed to stress. However, the efficiency of these antioxidants is not enough to reduce oxidative stress; therefore, we need to gain knowledge on mechanisms that can boost antioxidative metabolism. Furthermore, osmolytes are the other crucial components that maintain the cell redox state by functioning as an antioxidant on the one hand and maintaining osmotic balance on the other. ROS suppression could also be achieved through phytohormones, which activate signals for antioxidant defense and osmolytes accumulation. 

Nitric oxide and ABA are essential regulators for plants defense against stress. Iqbal et al. [49] reviewed the interaction between ABA and NO for heat tolerance and explored various common mechanisms in their interaction. In the present work, we have studied the effect of NO and ABA in alleviating heat stress individually and in combination, also studied how they regulate each other through the use of NO and ABA inhibitors. Nitric oxide is a gaseous signaling molecule that functions as a signaling molecule at low concentrations, whereas it causes nitro-oxidative stress at higher concentrations, causing cellular damage. Nitric oxide and ABA increase the antioxidant capacity under high temperature stress [56,97], suppress ROS, and maintain cellular redox homeostasis. The role of these hormones in regulating osmolytes has also been investigated [57,98,99]. We observed that both NO and ABA help in heat tolerance, and the ABA effect was depended on NO. However, when applied simultaneously, we observed maximum heat stress alleviation.

### 4.1. Role of NO and ABA in Reducing Photosynthesis Inhibition and Growth under Heat Stress 

Heat stress adversely affects the photosynthesis and growth of plants [12,13,70] by affecting the thylakoid membrane, chloroplast protein complexes, photosystem II, and activity of Rubisco [13,100]. In order to protect plants from heat stress, the photosynthetic efficiency of plants should be improved. Nitric oxide prevents chlorophyll loss and protects activity of PSII and photosynthesis under stress [12,101,102]. Treatment with SNP was found to maintain the Fv/Fm level while inhibiting F_0_ rise, and increased chlorophyll and Rubisco by overexpressing *NOA1* in rice [103]. The effect of NO in photosynthetic protection is concentration dependent; at a high concentration, it causes damage to the photosynthetic machinery in response to high temperature [70]. Similarly, a high NO concentration was found in the present study under heat stress. However, NO whensupplied exogenously under heat stress signaled for the reduction in oxidative stress through the increase in the antioxidant enzymes, which subsequently caused lower NO generation. Excess NO in *Pisum sativum* reduced photosynthesis probably by reversibly binding to the thylakoid membrane complex and restricting electron transport [104]. 

Nitric oxide crosstalk with plant abiotic-stress responses and stress regulators exists. In this study, its crosstalk with ABA has been discussed. It has been shown that ABA-responsive-element binding protein 9 (ABP9) improves photosynthesis under heat stress in *Arabidopsis* [105]. Increased production of ABA under heat stress increases activity of antioxidant enzymes and helps maintain plants’ water status through the regulation of stomata [56,106]. We observed that ABA supplementation under heat stress resulted in increased production of H_2_O_2_, leading to an increase in antioxidants to adjust to heat stress. The decrease in photosynthesis under heat stress in plants supplemented with ABA in comparison to the control could probably be due to increased H_2_O_2_ and partial stomatal closure supported by reduced stomatal conductance and intercellular CO_2_ concentration. The combined NO with ABA maximally increased the activity and expression of antioxidant enzymes and osmolytes production to scavenge ROS efficiently and improve photosynthesis above the control. It may be emphasized that NO with ABA restricted the ABA-mediated increase in H_2_O_2_ content. The effect of ABA on photosynthesis was found to be dependent on NO because inhibition of NO by cPTIO inhibited the observed increase in photosynthesis by ABA. This is also indicative of NO signaling downstream of ABA, while ABA inhibition had little effect on NO action. A study by Wang et al. [107] suggested that ABA signaling results in NO formation, which causes *S*-nitrosylation of *SnRK2.6* at a cysteine residue close to the kinase catalytic site and blocks the kinase activity resulting in ABA insensitivity in stomatal regulation.

Similar to the increase in photosynthetic trait, there was an increase in leaf area and plant dry mass with both NO, ABA, and their combination under heat stress. An increase in growth was observed in *Oryza sativa* plants when NO was applied exogenously at low concentrations and alleviated salt or heat stress [108]. In the callus of *Phragmites communis*, 0.2 mM SNP and *S*-nitrose-*N*-acetyl penicillamine reduced the adversities of heat and protected a reduction in growth and cell viability [27]. ABA also protects plant growth under stress and ABA signaling mutant *abi-1* exhibited increased TBARS content and reduced the survival rate under heat stress together with a reduction in fresh weight and plant diameter in response to heat stress [109]. However, the reports on NO and ABA interaction under heat stress are scanty. We have reported that when ABA-treated plants are supplied with NO, synergy in their action mediates the maximum increase in antioxidants and osmolytes to protect plants from the adversities of heat stress on photosynthesis.

### 4.2. Nitric Oxide and ABA Increased Osmolytes Accumulation and Antioxidant Enzyme Activity and Expression to Reduce Heat-Induced Oxidative Stress

In the present study, the decrease in oxidative stress was due to enhanced activity of the antioxidative enzymes SOD, CAT, APX, GR, and APX and GR gene expression. A similar decrease in H_2_O_2_ content and increase in antioxidant enzymes with ABA application under heat stress has been reported [110]. It has been shown that both ABA and H_2_O_2_ increased NO generation in mesophyll cells of maize leaves, and H_2_O_2_ was required for the ABA-induced NO generation [111]. In the present study also, ABA function was dependent on NO since inhibition of NO by NO scavenger substantially reduced the production of ABA-induced NO and activities of several antioxidant enzymes that were induced with ABA. In *Stylosanthes guianensis*, ABA-mediated NO generation was responsible for the increase in antioxidant enzymes; however, blocking the NO generation by specific inhibitors reduced the ABA-induced activity of antioxidants under heat stress [78].

The ameliorative effect of NO with ABA was also because of the increase in osmolytes accumulation. We studied the content of proline, GB, trehalose, and total soluble sugar in heat-stressed plants and found their content increased under heat stress, except for total soluble sugars, for which the content decreased. Application of either NO or ABA increased the content of all these osmolytes but maximally when they were applied in combination. Osmotic adjustment helps protect the cells against abiotic stresses, and it has an important role in regulating membrane fluidity and scavenging ROS. Proline, an osmoprotectant, protected *Vicia faba* under heat stress, and the increase in proline increased with an increase in NO [94]. Nitric oxide helped in mitigating oxidative stress by maintaining cellular redox homoeostasis by neutralizing ROS under heat stress [2]. Sharma et al. [112] reviewed phytohormonal regulation of osmolytes accumulation under different abiotic stress conditions. Under salt stress in wheat seedlings, NO increased the synthesis of endogenous ABA and acted downstream of ABA in ABA-induced proline accumulation [61]. Applying exogenous ABA to plants under drought stress significantly enhanced the sugar accumulation but decreased the starch content in leaves [113]. Its application increased the levels of sugar and related key enzymes to improve cold tolerance in winter wheat [114]. It was found that ABA enhanced sugar accumulation under saline conditions [115] and ABA and sucrose increased the survival rate of bromegrass cells under heat stress [116]. It has been reported that genotypes that maintain a higher accumulation of proline, GB, and expression of heat shock proteins, together with activity of antioxidant enzymes such as catalase, peroxidase, SOD, and GR, can tolerate high temperature efficiently through sustaining cellular physiology [117]. Nitric oxide also influences soluble sugar accumulation under heat stress. Supplementation of NO to heat-stressed plants reduced the stress-induced glucose accumulation by increasing its utilization for growth [70]. Exogenous NO with Ca^2+^ alleviated oxidative stress caused by high temperature through enhancement in osmolyte accumulation, antioxidants, and nitrate reductase (NR) activity [118]. In tomato, SNP supplementation reduced oxidative stress via increased synthesis of compatible solutes and antioxidative enzymes together with increased activity of Rubisco, NR activity, total structural carbohydrate, and photosynthetic pigments in tomato [60]. The role of GB in heat tolerance is advocated by various studies [119,120]. ABA was found to affect GB by regulating the activity of BADH via modulating the phosphorylation process of important receptors such as SnRK2 [121]. In chickpea, ABA was found to increase GB and proline together with trehalose content to impart heat tolerance [59]. Similarly, SNP increased GB and proline content in *S. lycopersicum* seedlings for heat stress mitigation together with Ca^2+^ [60]. GB content was found to increase with a NO donor, while a NO inhibitor blocked its formation, supporting the role of NO in GB formation [122]. Trehalose is a non-reducing disaccharide, which accumulated in *Arabidopsis* plants under heat stress [123] and acted as a ROS scavenger in heat-stressed wheat plants [124]. Trehalose regulates water-use efficiency and stomatal movement in most plants and is vital for sustaining growth under stressful situation [125]. Li et al. [64] have reported that trehalose helps in the stabilization of dehydrated enzymes, proteins, and membrane lipids and protects biological structures from damage because of its function as an osmoprotectant during desiccation. Trehalose accumulation increased in the present study under heat stress and further with both ABA and NO application. Trehalose accumulation increased with NO in *P. eryngii* var. *tuoliensis* under heat stress [126]. Osmoprotectants such as proline, GB, and trehalose increase under heat stress and function as stress signaling molecules to protect denaturation of enzymes, membrane stabilization, and protection of photosynthetic pigments, this through their role as antioxidants to scavenge deleterious ROS and as osmolytes to maintain osmotic homeostasis [127,128]. The increase in these osmolytes in our present study is also indicative of the increase in tolerance potential of wheat with NO and ABA under heat stress.

### 4.3. The Interaction of NO and ABA and Their Production 

The literature has shown an increase in NO and ABA content in various plant species against high-temperature treatments [31,106]. Nitric oxide-mediated regulation of ABA biosynthesis for water stress was reported by Xing et al. [129]. In the present study, NO supplementation decreased the NO content even under heat stress. Such a decrease has also been reported earlier by Iqbal et al. [70]. It also decreases the ABA content compared to heat treatment. The reduction in ABA was optimum in NO and ABA combined treatment, which maximally alleviated stress. Reports are suggestive of NO and ABA interaction, and it has recently been reviewed [49]. Zhang et al. [130] reported increased NO formation in transgenic tobacco over-expressing *SgNCED1* together with increased contents of ABA, H_2_O_2_, and activities of antioxidant enzyme to impart drought and salt stress tolerance. They reported that NO acts as a downstream signaling element in the ABA signaling pathway, and a reduction in NO production or its scavenging decreases or, in some cases, even eliminates ABA responses. However, ABA inhibition caused no effect on exogenous NO-mediated responses. In contrast, NO was not always required during the ABA signaling cascades as during stomatal closure [131] or light-induced stomatal opening inhibition [132]. The regulation of antioxidant enzymes maintains adjustment of the proper NO/ROS balance by S-nitrosylation [133,134], and this is an absolute requirement for stress-induced ROS. Heat stress increased the content of both NO and ABA; however, their exogenous application lowered the heat stress and also their content. Further, since ABA has been reported to increase the H_2_O_2_ content and consequently antioxidant activity, the H_2_O_2_ content was comparatively higher with ABA treatment compared to NO treatment.

## 5. Conclusions

It may be concluded that NO and ABA reduced the heat stress impacts on photosynthesis and growth, independently and maximally, with their combined application through regulation of activity and expression of antioxidant enzymes and osmolytes accumulation. The greatest benefit was obtained with the combined application of NO and ABA. However, NO was better than ABA through a maximum increase in activity of antioxidant enzymes and osmolytes accumulation. The response of plants to ABA was NO dependent. The inhibition of NO significantly reduced the production of ABA-induced NO and activity of antioxidant enzymes that were induced with ABA. The adaptation of plants against heat stress by ABA can be enhanced by the addition of NO. 

## Figures and Tables

**Table 1:** Plant length, leaf area, plant fresh weight, and plant dry weight of wheat (*Triticum aestivum* L. var. WH 542) treated with 100 µM SNP and/or ABA in the presence (40 °C) or absence (25 °C) of heat stress at 30 days after sowing (DAS). The combined NO and ABA treatment under heat stress was also subjected to 100 µM cPTIO or 80 µM fluridone at 30 DAS. Data are presented as the treatment mean ± SE (*n* = 4). In each column, data followed by the same letter are not significantly different by Tukey test at *p* < 0.05. ABA, abscisic acid; cPTIO, 2-4-carboxyphenyl-4,4,5,5-tetramethylimidazoline-1-oxyl-3-oxide; Flu, fluridone; HT, heat stress; SNP, sodium nitroprusside.

Treatments	Plant Length (cm)	Leaf Area (cm^2^ Plant^−1^)	Plant Fresh Weight	Plant Dry Weight
			(g Plant^−1^)	
Control	47 ± 2.35d	27.2 ± 1.2d	2.8 ± 0.14d	0.91 ± 0.042d
HT	37 ± 1.82g	18.3 ± 0.84g	2.1 ± 0.11g	0.44 ± 0.017g
SNP	52 ± 1.02b	33.1 ± 1.28b	3.2 ± 0.16b	1.29 ± 0.060b
ABA	50 ± 2.40c	29.4 ± 1.72c	3.1 ± 0.15c	1.09 ± 0.051c
SNP + ABA	55 ± 1.11a	38.8 ± 1.99a	3.6 ± 0.18a	1.44 ± 0.072a
HT + SNP	48 ± 2.36cd	28.2 ± 1.65cd	2.6 ± 0.13d	0.98 ± 0.054d
HT + ABA	44 ± 2.21e	25.3 ± 1.23e	2.4 ± 0.12e	0.73 ± 0.044e
HT + SNP + ABA	51 ± 2.49b	32.2 ± 1.54b	2.9 ± 0.14b	1.22 ± 0.065b
HT + SNP + ABA + Flu	47 ± 2.32d	27.3 ± 1.11d	2.7 ± 0.13cd	1.03 ± 0.046cd
HT + SNP + ABA + cPTIO	40 ± 1.98f	19.6 ± 0.98f	2.2 ± 0.11f	0.53 ± 0.033f

**Table 2:** Net photosynthesis, stomatal conductance, intercellular CO_2_ concentration, chlorophyll content, maximum efficiency of PSII, and ribulose 1,5 bisphosphate carboxylase/oxygenase (Rubisco) activity of wheat (*Triticum aestivum* L. var. WH 542) leaves treated with 100 µM SNP and/or ABA in the presence (40 °C) or absence (25 °C) of heat stress at 30 DAS. The combined NO and ABA treatment under heat stress was also subjected to 100 µM cPTIO or 80 µM fluridone at 30 days after sowing (DAS). Data are presented as the treatment mean ± SE (*n* = 4). In each column, data followed by the same letter are not significantly different by Tukey test at *p* < 0.05. ABA, abscisic acid; cPTIO, 2-4-carboxyphenyl-4,4,5,5-tetramethylimidazoline-1-oxyl-3-oxide; Flu, fluridone; HT, heat stress; SNP, sodium nitroprusside.

Treatments	Net Photosynthesis (µmol CO_2_ m^−2^ s^−1^)	Stomatal Conductance (mmol CO_2_ m^−2^ s^−1^)	Intercellular CO_2_ Concentration (µmol mol^−1^)	Chlorophyll Content (SPAD Value)	Maximum Quantum Yield Efficiency of PSII (Fv/Fm)	Rubisco (µmol CO_2_ mg^−1^ Protein min^−1^)
Control	11.2 ± 0.78d	362 ± 16.1d	211 ± 10.7d	30.3 ± 1.6d	0.72 ± 0.040d	38.9 ± 2.1d
HT	06.2 ± 0.64g	259 ± 14.3g	119 ± 6.47g	19.1 ± 1.4g	0.60 ± 0.030g	25.2 ± 1.6g
NO	16.1 ± 1.09b	433 ± 18.9b	354 ± 16.9b	44.2 ± 2.8b	0.88 ± 0.050b	53.4 ± 2.6b
ABA	14.6 ± 0.84c	416 ± 17.8c	319 ± 12.8c	40.4 ± 2.1c	0.75 ± 0.480c	47.8 ± 2.4c
NO + ABA	18.2 ± 0.99a	456 ± 20.6a	376 ± 17.19a	49.1 ± 3.4a	0.97 ± 0.060a	57.7 ± 2.8a
HT + NO	12.5 ± 0.87d	356 ± 16.8d	228 ± 11.7d	31.8 ± 1.8d	0.73 ± 0.041d	40.2 ± 2.3d
HT + ABA	09.8 ± 0.84e	328 ± 15.2e	191 ± 10.8e	28.6 ± 2.1e	0.66 ± 0.036e	32.7 ± 2.2e
HT + NO + ABA	15.4 ± 0.94c	409 ± 18.2c	326 ± 13.3c	39.3 ± 1.9c	0.87 ± 0.050c	48.1 ± 2.4c
HT + NO + ABA + Flu	11.9 ± 0.83d	357 ± 15.4d	203 ± 09.6d	31.2 ± 1.8d	0.77 ± 0.440d	40.6 ± 2.2d
HT + NO + ABA + cPTIO	07.3 ± 0.77f	302 ± 13.9f	152 ± 07.3f	25.4 ± 1.1f	0.66 ± 0.035f	32.5 ± 1.8f

**Table 3:** Activity of superoxide dismutase (SOD), catalase (CAT), ascorbate peroxidase (APX), and glutathione reductase (GR) of wheat (*Triticum aestivum* L. var. WH 542) leaves treated with 100 µM SNP and/or ABA in the presence (40 °C) or absence (25 °C) of heat stress at 30 days after sowing (DAS).The combined NO and ABA treatment under heat stress was also subjected to 100 µM cPTIO or 80 µM fluridone at 30 days after sowing (DAS). Data are presented as the treatment mean ± SE (*n* = 4). In each column, data followed by the same letter are not significantly different by Tukey test at *p* < 0.05. ABA, abscisic acid; cPTIO, 2-4-carboxyphenyl-4,4,5,5-tetramethylimidazoline-1-oxyl-3-oxide; Flu, fluridone; HT, heat stress; SNP, sodium nitroprusside.

Treatments	SOD	CAT	APX	GR
	(U min^−1^ mg^−1^ protein)			
Control	7.69 ± 0.41f	122 ± 09.2g	2.2 ± 0.23f	2.29 ± 0.18f
HT	11.9 ± 0.61e	146 ± 09.9f	3.7 ± 0.33e	3.72 ± 0.21e
SNP	12.9 ± 0.84d	161 ± 10.4d	4.4 ± 0.48d	4.11 ± 0.27d
ABA	12.3 ± 0.69d	142 ± 10.7e	3.3 ± 0.31e	3.71 ± 0.24e
SNP + ABA	15.1 ± 1.21c	174 ± 11.2c	5.7 ± 0.55c	4.90 ± 0.34c
HT + SNP	18.3 ± 0.99b	191 ± 12.3b	6.7 ± 0.63b	5.80 ± 0.43b
HT + ABA	15.4 ± 1.29c	169 ± 11.9c	5.3 ± 0.43c	4.70 ± 0.45c
HT + SNP + ABA	20.7 ± 1.38a	206 ± 13.8a	8.2 ± 0.71a	6.40 ± 0.42a
HT + SNP + ABA + Flu	17.7 ± 1.17b	181 ± 12.4b	6.1 ± 0.39b	5.70 ± 0.51b
HT + SNP + ABA + cPTIO	12.0 ± 0.72de	149 ± 11.5f	3.4 ± 0.29e	3.89 ± 0.14e

## Data Availability

Data are contained within the article and Supplementary Materials.

## Supplementary Materials

The following supporting information can be downloaded at: https://www.mdpi.com/article/10.3390/antiox11020372/s1, Details of the Material and Methods are provided in the Supplementary Files; Table S1: Primer pairs used for quantitative RT-PCR.
