# Supplementary material for: Nitric Oxide and Abscisic Acid Mediate Heat Stress Tolerance through Regulation of Osmolytes and Antioxidants to Protect Photosynthesis and Growth in Wheat Plants
Source: Antioxidants (Basel). 2022 Feb 12;11(2):372. doi: 10.3390/antiox11020372 (PMC8869392; doi:10.3390/antiox11020372)
Supplement: Supplementary file 1 [file antioxidants-11-00372-s001.zip › antioxidants-1593037-supplementary.pdf]

antioxidants

MDPI

Article

Nitric Oxide and Abscisic Acid Mediate Heat Stress Tolerance through Regulation of Osmolytes and Antioxidants to Protect Photosynthesis and Growth in Wheat Plants

Noushina Iqbal [1], Zebus Sehar [2], Mehar Fatma[2], Shahid Umar[1]*, Adriano Sofo[3]* and Nafees A. Khan [2,*]

[1] Department of Botany, Jamia Hamdard, New Delhi 110062, India; naushina.iqbal@gmail.com (N.I.); sumer@jamiahamdard.ac.in (S.U.)

[2] Plant Physiology and Biochemistry Laboratory, Department of Botany, Aligarh Muslim University, Aligarh 202002, India; seharzebus5779@gmail.com (Z.S.); meharfatma30@gmail.com (M.F.); naf9.amu@gmail.com (N.A.K.)

[3] Department of European and Mediterranean Cultures: Architecture, Environment, Cultural Heritage (DiCEM), University of Basilicata, Via Lanera, 75100, Matera, Italy; adriano.sofo@unibas.it (AS)

* Correspondence: sumer@jamiahamdard.ac.in, adriano.sofo@unibas.it, naf9.amu@gmail.com

Citation: Iqbal, N.; Sehar, Z.; Fatma, M.; Umar, S.; Sofo, A.; Khan, N.A. Nitric Oxide and Abscisic Acid Mediate Heat Stress Tolerance through Regulation of Osmolytes and Antioxidants to Protect Photosynthesis and Growth in Wheat Plants. *Antioxidants* **2022**, *11*, 372. https://doi.org/10.3390/antiox11020372

Academic Editor: Stanley Omaye

Received: 26 January 2022
Accepted: 11 February 2022
Published: 12 February 2022

Publisher's Note: MDPI stays neutral with regard to jurisdictional claims in published maps and institutional affiliations.

Copyright: © 2022 by the authors. Licensee MDPI, Basel, Switzerland. This article is an open access article distributed under the terms and conditions of the Creative Commons Attribution (CC BY) license (https://creativecommons.org/licenses/by/4.0/).

Supplementary information

Photosynthetic characteristics measurements

The fully expanded third leaf of each treatment was taken for gas exchange measurements. Measurements for net photosynthesis (P_N), stomatal conductance (gs), intercellular CO_2 concentration (Ci) were taken on an Infrared Gas Analyzer (CID-340, Photosynthesis System, Bio-science, Washington, USA). All the measurements were taken between 11.00 and 12.00 h at atmospheric CO_2 concentration of 380 ± 5 μmol mol^{-1}, relative humidity of 70%, photosynthetic active radiation of 780 μmol m^{-2} s^{-1}, and air temperature at 28°C. Chlorophyll content was measured with SPAD chlorophyll meter (SPAD 502 DL PLUS, Konica Minolta, Japan).

The maximal efficiency of photosystem II (PSII), as given by Fv/Fm, was determined with a chlorophyll fluorometer (Junior-PAM, Heinz Walz, GmbH, Effeltrich, Germany). For measurement, fully expanded uppermost leaves were taken. The initial fluorescence (Fo) was determined by exposure of dark-adapted leaves to photosynthetically active photon flux density (PPFD) of (0.1 μmol photons m^{-2} s^{-1}), while maximum fluorescence (Fm) was obtained from saturating pulse (>6000 μmol photons m^{-2} s^{-1}). The Fm and variable fluorescence (Fv), given as (Fm–Fo) were assessed in the leaves dark-adapted for 30 min using leaf clips. Maximum efficiency of PSII was estimated by the calculation: Fv/Fm = (Fm–Fo)/Fm.

Rubisco activity

Fresh leaf samples (1.0 g) were homogenized in ice-cold extraction buffer containing 0.25 M Tris-HCl (pH 7.8), 0.05 M $MgCl_2$, 0.0025 M EDTA, and 37.5 mg dithiothreitol (DTT) using a pre-chilled mortar and pestle. The homogenate was centrifuged at 10,000×g for 10 min at 4°C, and the supernatant was used to measure enzyme activity. The reaction mixture (3.0 mL) contained 100 mM Tris-HCl (pH 8.0), 40 mM $NaHCO_3$, 10 mM $MgCl_2$, 0.2 mM NADH, 4 mM ATP, 5 mM DTT, 1U of glyceraldehyde 3-phosphodehydrogenase, 1U of 3-phosphoglycerate kinase, and 0.2

mM ribulose 1,5-bisphosphate (RuBP). Protein was estimated using bovine serum albumin as standard.

Content of hydrogen peroxide (H_2O_2) and lipid peroxidation

To determine the H_2O_2 content, fresh leaf tissue (500 mg) was homogenized in ice-cold 200 mM perchloric acid ($HClO_4$), followed by centrifugation at 1200 × g for 10 min. Later the supernatant was neutralized with 4 M KOH. Homogenate was further centrifuged at 500 × g for 3 min for the removal of insoluble potassium perchlorate. The reaction mixture (1.5 mL) contained 1 mL of the eluate, 80 µL of 3-methyl-2-benzothiazoline hydrazone, 400 µL of 12.5 mM 3-(dimethylamino) benzoic acid in 0.375 M phosphate buffer (pH 6.5), and 20 µL of peroxidase (0.25 unit). The reaction was initiated with the addition of peroxidase at 25°C.The increase in absorbance was estimated at 590 nm on a spectrophotometer.

Lipid peroxidation was determined by measuring thiobarbituric acid reactive substances (TBARS) content. Fresh leaf samples (500 mg) were ground in 0.25% 2-thiobarbituric acid (TBA) in 10% trichloroacetic acid (TCA) using mortar and pestle. The mixture was heated at 95°C for 30 min and rapidly cooled on ice bath. Then, mixture was allowed to centrifuge at 10,000 × g for 10 min. To one mL of the supernatant 4.0 mL of 20% TCA containing 5% TBA was added. The absorbance of the supernatant was measured at 532 nm and the absorbance of the same at 600 nm was subtracted to compensate for non-specific turbidity. The content of TBARS was computed using the extinction coefficient (155 mM^{-1} cm^{-1}).

Activity of antioxidant enzymes

Assay of activity of antioxidant enzymes

Fresh leaf tissue (200 mg) was collected from the top-most leaves and ground rapidly in ice-cold extraction buffer containing potassium-phosphate buffer (100 mM, pH 7.0), 0.05% (v/v) Triton X-100 and 1% (w/v) polyvinylpyrrolidone (PVP). The homogenate was centrifuged at 15,000×*g* for 20 min at 4°C. The supernatant obtained after centrifugation was used for the assay of enzymes. Protein was estimated according to protocol described by Bradford utilizing Bovine serum albumin as a protein standard.

Superoxide dismutase (SOD)

Briefly, for the SOD assay, 5.0 Ml of reaction mixture contained 5 mM HEPES (pH 7.6), 0.1 mM EDTA, 50 mM Na_2CO_3 (pH 10.0), 13 mM methionine, 0.025% (v/v) Triton X-100, 63 µmol NBT, 1.3 µmol riboflavin was mixed with enzyme extract. The reactants were then placed in a bright light (360 µmol m^{-2} s^{-1}) for 15 min and the control set was not illuminated to correct for background absorbance. One unit of SOD is defined as the amount of enzyme that inhibited the NBT reduction by 50% at 560 nm.

Catalase (CAT)

A 1.5 mL of reaction mixture consisting of 13.2 mM H_2O_2 in 50 mM phosphate buffer (pH 7.0) and 0.1 mL of enzyme extract and a control set was also illuminated for correcting the background absorbance.

Ascorbate peroxidase (APX)

The assay mixture (1.0 mL) contained phosphate buffer (50 mM, pH 7.0), 0.1 mM EDTA, 0.5 mM ascorbate, 0.1 mM H_2O_2, and enzyme extract. Final volume was observed at 290 nm for 1 min using spectrophotometer. A decrease in absorbance was observed as soon as the reaction was started by adding H_2O_2. APX activity was computed with the extinction coefficient of 2.8 mM^{-1} cm^{-1}. One unit of enzyme is the amount necessary to decompose 1 μmol of substrate per min at 25°C.

Glutathione reductase (GR)

The reaction mixture (3.0 mL) contained phosphate buffer (25 mM, pH 7.8), 0.5 mM GSSG, 0.2 mM NADPH, and the enzyme extract. The reaction started with the addition of GSSG, and the absorbance showed a decreasing trend. The activity of GR was quantified by using the extinction coefficient of 6.2 mM^{-1} cm^{-1}. One unit of enzyme is the amount necessary to decompose 1 μmol of NADPH min^{-1} at 25°C.

Total soluble sugar content

Leaf samples were oven-dried at 80°C and ground to a fine powder. Approximately 100 mg of the dried sample was extracted using 10 mL of 80% ethanol and kept in a water bath at 80-85°C for 30 min. The extract was centrifuged and the supernatant was transferred to a 100 mL volumetric flask, the extraction was repeated three times. Alcohol extract was evaporated on a water bath at 80-85°C. All the three supernatants were pooled in the flask following by addition of distilled water to 100 mL. Aliquot of the extract was used for determination of soluble sugars with anthrone reagent and the absorbance of reaction mixture was monitored at 630 nm using a spectrophotometer.

Trehalose content

The leaves (1.0 g) were homogenized in 5 mL of 80% (v/v) hot ethanol and centrifuged at 11,500 × *g* for 20 min. The supernatant was dried at 80 °C followed by resuspension in 5 mL distilled water. The solution (100 μL) was mixed with 150 μL 0.2 N H_2SO_4 and boiled at 100°C for 10 min to hydrolyze any sucrose or glucose-1-phosphate and then chilled on ice. NaOH (0.6 N, 150 μL) was added to the above mixture and boiled for 10 min to destroy reducing sugars, and then chilled again. To the above mixture, 2.0 mL of anthrone reagent (0.2 g anthrone per 100 ml of 95% H_2SO_4) was added and boiled for 10 min to develop a colour, and then chilled again. The absorbance was recorded at 630 nm, and trehalose concentration was calculated as micromoles per gram fresh weight (FW) using a standard curve developed with commercial trehalose.

RNA isolation and cDNA synthesis

Total RNA was isolated from rice leaves using TRIzol reagent (Ambion, Life Technologies, Austin, TX, USA) according to the manufacturer's instructions. The extracted RNA was quantified using a Nanodrop spectrophotometer (Thermo Scientific, Waltham,

MA, USA). To ensure the integrity of the RNA, each sample was run on an agarose formaldehyde gel [115]. For both the control and treated samples, first-strand cDNA was made from 1 μg of total RNA and a reaction mixture containing 20 U/μL Moloney murine leukemia virus reverse transcriptase (MuMLV) (Fermentas, Waltham, MA, USA), incubated at 42 °C for 50 min and at 70 °C for 10 min. The reverse transcription reaction was carried out using 2.5 μM Oligo (dT) 18 primer (Fermentas, USA) and 10 mM dNTPs. Primers for gene expression analysis were designed using online primer designing software (IDT), and the cDNA sequences of selected genes were obtained from NCBI.

Quantitative real-time PCR analysis

Real-time PCR (RT-PCR) was performed in 96-well reaction plates (Roche, Mannheim, Germany) containing a 20-μL reaction mixture of 10X reaction buffer, 2 mM dNTPs, 1 mM $MgCl_2$, 0.35 μM each of forward and reverse primers, 1 μL SYBR Green (10X), 10 μg cDNA template, and 5 U Taq polymerase on a thermal cycler (Lightcycler 480 II, Roche, Germany). All quantifications were normalized to an actin DNA fragment amplified by β-actin forward and β-actin reverse primers, and the actin gene was used as an internal control for evaluating the per-gene efficiency of RT-PCR. PCR cycling conditions were as follows: denaturation at 95 °C for 3 min, 40 cycles of 95 °C (20 s), 66 °C (1 min) and 72 °C (1 min), and a final extension at 72 °C for 5 min. The amplified product was resolved on a 1.2% agarose gel. Amplicon specificity was verified by melting curve analysis (60 to 95 °C). All reactions were performed as three biological replicates (with three technical replicates of each), using gene-specific primers and actin primers as an internal control. The primer pairs used for quantitative RT-PCR are listed in Supplementary Table S1. The results were presented as the expression of the gene of interest in relation to the internal control in the treated sample compared with corresponding values in the untreated control.

Table S1. Primer pairs used for quantitative RT-PCR.

S.No.	Gene	Gene ID	Forward primer	Reverse primer
1	GR	123146096	GCCATGTGTGGACCAGATGC	GCAGAAGGGTGGATCCCGAC
2	APX	542880	GAGTGTCCGGAGGAGGGGAG	CTTTCCCCAGCCACTCCTGT
**Reference gene primer sequences used for quantitative RT-PCR**				
1	Actin	123048645	GACTGCCAAGACCAGCTCC	CTTCCTAATATCCACGTCGCAC
